# Stable isotope probing of hypoxic toluene degradation at the Siklós aquifer reveals prominent role of *Rhodocyclaceae*

**DOI:** 10.1093/femsec/fiy088

**Published:** 2018-05-14

**Authors:** András Táncsics, Anna Róza Szalay, Milan Farkas, Tibor Benedek, Sándor Szoboszlay, István Szabó, Tillmann Lueders

**Affiliations:** 1 Regional University Center of Excellence in Environmental Industry, Szent István University, Páter K. u. 1., 2100 Gödöllő, Hungary; 2 Institute of Groundwater Ecology, Helmholtz Zentrum München, German Research Center for Environmental Health, Ingolstädter Landstr. 1., 85764 Neuherberg, Germany; 3 Department of Environmental Safety and Ecotoxicology, Szent István University, Páter K. u. 1., 2100 Gödöllő, Hungary

**Keywords:** biodegradation, oxygen limitation, DNA-stable isotope probing, subfamily I.2.C extradiol dioxygenase (C23O), groundwater, *Rhodocyclaceae*

## Abstract

The availability of oxygen is often a limiting factor for the degradation of aromatic hydrocarbons in subsurface environments. However, while both aerobic and anaerobic degraders have been intensively studied, degradation betwixt, under micro- or hypoxic conditions has rarely been addressed. It is speculated that in environments with limited, but sustained oxygen supply, such as in the vicinity of groundwater monitoring wells, hypoxic degradation may take place. A large diversity of subfamily I.2.C extradiol dioxygenase genes has been previously detected in a BTEX-contaminated aquifer in Hungary. Older literature suggests that such catabolic potentials could be associated to hypoxic degradation. Bacterial communities dominated by members of the *Rhodocyclaceae* were found, but the majority of the detected C23O genotypes could not be affiliated to any known bacterial degrader lineages. To address this, a stable isotope probing (SIP) incubation of site sediments with ^13^C_7_-toluene was performed under microoxic conditions. A combination of 16S rRNA gene amplicon sequencing and T-RFLP fingerprinting of C23O genes from SIP gradient fractions revealed the central role of degraders within the *Rhodocyclaceae* in hypoxic toluene degradation. The main assimilators of ^13^C were identified as members of the genera *Quatrionicoccus* and *Zoogloea*, and a yet uncultured group of the *Rhodocyclaceae*.

## INTRODUCTION

The contribution of microbes to the removal of BTEX compounds (benzene, toluene, ethylbenzene and xylenes) from groundwater ecosystems has been intensively investigated over the last decades (Lueders 2017). However, most studies have addressed either strictly aerobic or anaerobic degradation and degraders, often by using enriched or pure cultures in highly artificial laboratory systems. In subsurface ecosystems, the availability of oxygen is often restricted, with hydrocarbon contamination causing microoxic or anoxic conditions even in shallow aquifers. Under oxic conditions, genes for aromatic ring-cleavage dioxygenase enzymes are key to the degradation of monoaromatic compounds (El-Naas, Acio and El Telib 2014). Aerobic degraders use oxygen not only for respiration but also as a cosubstrate for these enzymes. However, Kukor and Olsen (1996) suggested that a specific group of extradiol dioxygenases (subfamily I.2.C) was adapted to environments with low oxygen concentrations, hinting at their role in ring-cleavage reactions in what they called “oxygen-requiring, but nitrate-enhanced” hypoxic degradation. Nevertheless, it has to be noted that ring-cleaving dioxygenases belonging to the same subfamily may show different oxygen affinities as this was observed in case of chlorocatechol 1,2-dioxygenases (Balcke *et al*. 2008).

Previous investigations of an oxygen-limited BTEX-contaminated shallow aquifer in Siklós, Hungary have revealed a notable diversity of catechol 2,3-dioxygenase (C23O) genes encoding subfamily I.2.C-type extradiol dioxygenases at the site (Táncsics *et al*. 2012, 2013). It was also shown that the bacterial community at this site was dominated by microorganisms affiliated to the *Comamonadaceae* and *Rhodocyclaceae*. Both betaproteobacterial lineages are known to harbor aromatic hydrocarbon degraders. However, *Comamonadaceae*-affiliated degraders (e.g. members of the genera *Acidovorax, Comamonas, Delftia, Diaphorobacter, Hydrogenophaga, Polaromonas* and *Variovorax*) utilize BTEX-compounds only aerobically and usually harbor subfamily I.2.C-type C23Os (Parales 2010). On the other hand, many *Rhodocyclaceae*-affiliated degraders degrade aromatic hydrocarbons under anaerobic conditions (members of the genera *Azoarcus, Dechloromonas*and*Thauera*) (Weelink, van Eekert and Stams 2010). Recently, however, some members of the genus *Zoogloea* and the type species of the genus *Rugosibacter* have been identified as aerobic hydrocarbon degraders (Jechalke *et al*. 2013; Farkas *et al*. 2015; Corteselli, Aitken and Singleton 2017), showing that members of the *Rhodocyclaceae* can also have a role in aerobic degradation processes.

Although I.2.C-type C23O genes can be abundant in hypoxic BTEX-contaminated groundwater ecosystems (Táncsics *et al*. 2012; Benedek *et al*. 2016), the majority of these genotypes cannot yet be linked to cultured bacteria. The aim of the present study was to identify, by means of DNA stable isotope probing (DNA-SIP), key degraders and associated I.2.C-type C23O genes active in toluene degradation under oxygen-limited conditions. For this, fresh sediment samples taken from the bottom of a monitoring well in the center of the BTEX plume at the Siklós site were incubated in microcosms under amendment of ^13^C_7_-toluene and a repeated replenishment of <0.5 mg/l oxygen as electron acceptor and as co-substrate for aromatic-ring-hydroxylating and ring-cleaving dioxygenases. Key bacteria labelled during microaerobic toluene degradation were identified as members of the *Rhodocyclaceae* and their catabolic genotypes were unraveled. This study provides new evidence that the known diversity of hypoxic degraders of BTEX compounds is still incomplete.

## MATERIALS AND METHODS

### Sampling site and sample acquisition

Sampling was performed at an intensively studied BTEX-contaminated aquifer (Táncsics *et al*. 2012, 2013; Farkas *et al*. 2017) in Siklós, Hungary, in April 2015. Sediment samples were taken from the bottom of a monitoring well at 6 m below ground surface in the center of the contaminant plume (well ST-2). Well sludge and hypoxic groundwater was retrieved by suction pumping (Gardena, Ulm, Germany) into a clean 10-L plastic jerrycan. After settling for ∼20 min, sediment sludge was dispensed into sterile 1-L glass bottles filled with *in situ* groundwater to minimize atmospheric exposure and transported to the laboratory under cooling.

### Incubation of sediments

Triplicates of 5g_ww_ homogenously mixed sediment material were transferred into sterile 100-mL serum bottles containing 50 mL of artificial groundwater medium (Winderl *et al*. 2010). To increase microbial activity 5 µm cAMP was added to the medium (Bruns, Cypionka and Overmann 2002). Bottles were sparged aseptically with N_2_/CO_2_ (80:20, v/v) for 10 min, after which the desired volume of sterile (0.2 µm-pore-size-filtered) air was injected into the bottles through gastight viton rubber stoppers. Dissolved oxygen concentration in the bottles was set to 0.5 mg/L, and kept between 0.5 and 0 mg/L throughout the experiment. Oxygen was replenished once every 24 h. A 5 µL of either non-labeled (^12^C) or fully labeled (^13^C_7_) toluene (Sigma-Aldrich, St. Louis, MO, USA) were injected to the microcosms. Abiotic control bottles (autoclaved three times) amended with unlabelled toluene were also prepared to exclude abiotic toluene loss or redox reactions. The bottles were incubated at 16°C in a rotary shaker at 145 rpm for over 7 d.

### Process measurements

The concentration of dissolved oxygen in the liquid phase of the microcosms was measured by using planar oxygen sensor spots and a Fibox 3 Oxygen Meter (PreSens, Regensburg, Germany). At each sampling spot, dissolved oxygen concentrations were registered every second during 1 min, and the results were displayed by using the OxyView-PST3 software (V7.01, PreSens). Toluene concentrations were determined by headspace analysis on an ISQ Single Quadrupole GC-MS (Thermo Fischer Scientific, Waltham, MA , USA) via a SLB-5ms fused silica capillary column (Sigma-Aldrich). The oven temperature was set to 40°C for 3 min, then ramped at a rate of 20°C/min to 190°C, and held for 1 min. The mass spectrometer (MS) was operated at 250°C in full scan mode.

### Nucleic acid extraction and ultracentrifugation

Sediments were collected from sacrificed microcosms after 3 and 7 d of incubation by centrifugation at 2360 *g* at 4°C for 10 min using a Rotanta 460 R (Hettich, Tuttlingen, Germany). Sludge pellets were frozen immediately at −80°C and DNA was extracted by using the RNA PowerSoil Total RNA Isolation Kit (MoBio, Carlsbad, CA, USA) in combination with the RNA PowerSoil DNA Elution Accessory Kit (MoBio). DNA samples were stored frozen at −80°C until downstream analyses. Approximately 1 µg of Qubit-quantified (Invitrogen, Paisley, UK) DNA extract was loaded onto a gradient medium of CsCl (average density 1.71 g/mL, Calbiochem, Darmstadt, Germany) in gradient buffer (0.1 M Tris-HCl at pH 8, 0.1 M KCl, 1mM EDTA) and centrifuged (180 000 *g*, ∼68 h) as previously described (Lueders 2015). A total of 12 fractions from each gradient were collected from ‘heavy’ to ‘light’ using a Perfusor V syringe pump (B. Braun, Melsungen, Germany). Refractometric measurement of fraction buoyant densities (BD) and the recovery of DNA from gradient fractions were performed as described (Lueders 2015).

### qPCR, T-RFLP fingerprinting and amplicon sequencing

DNA samples recovered from the CsCl gradient fractions were analyzed by qPCR targeting bacterial 16S rRNA gene as described (Kunapuli, Lueders and Meckenstock 2007; Pilloni *et al*. 2011). Eight DNA fractions (from 3rd to 10th) of each gradient were selected for bacterial 16S rRNA gene-targeted terminal restriction fragment length polymorphism (T-RFLP) fingerprinting, together with total DNA extracts of the inoculum. FAM labeled amplicons were generated with the primers Ba27f (5΄FAM-AGA GTT TGA TCM TGG CTC AG-3΄) and 907r (5΄-CCG-TCA-ATT-CCT-TTG-AGT-TT-3΄) similarly as described earlier (Pilloni *et al*. 2011). Amplicons were restricted using *Rsa*I, separated by capillary electrophoresis and electropherograms were evaluated as reported (Pilloni *et al*. 2011).

DNA extracts were also subjected to I.2.C-tpye C23O gene T-RFLP fingerprinting. VIC labeled amplicons were generated with the primers XYLE3F (5΄VIC- TGY TGG GAY GAR TGG GAY AA-3′) and XYLE3R (5′-TCA SGT RTA SAC ITC SGT RAA-3′) in a ProFlex PCR System (Life Technologies, Carlsbad, CA, USA) applying cycling conditions and PCR chemistry as reported (Táncsics *et al*. 2013). Amplicons were digested with *Alu*I, then electropherograms were generated and analyzed as described earlier (Farkas *et al*. 2017).

Non-density-resolved total DNA extracts from the inoculum and selected gradient fractions were also subjected to 16S rDNA amplicon pyrosequencing.

Bacterial 16S rRNA gene amplicon pyrosequencing was performed using a unidirectional sequencing approach as described (Zhang and Lueders 2017). Barcoded amplicons for multiplexing were prepared using the primers Ba27f (5΄-aga gtt tga tcm tgg ctc ag-3΄) and Ba907r (5΄-ccg tca att cmt ttr agt t-3΄) extended with the respective Lib-L adapters, key sequence and a multiplex identifier (MID) attached to the forward primer as recommended for the 454 GS FLX+ protocol (Roche, Basel, Switzerland). PCR amplification conditions were the same as described before (Karwautz and Lueders 2014). Amplicons were visualized with gel electrophoresis in a 1.5% agarose gel. Cleanup of the amplicons was done with a PCRextract kit (5Prime, Hamburg, Germany) according to the manufacturer’s protocol. Quality of single amplicons was checked for primer dimer contamination and correct fragment size using the Bioanalyzer2100 (Agilent, Santa Clara, CA, USA) loading High Sensitivity DNA assay chips (Agilent), as described by the manufacturer. One multiplexed amplicon pool (consisting of 20 amplicon libraries) was prepared in equimolar amounts (5*10^9^ molecules µl^−1^) of barcoded amplicons as quantified by the Quant-iT PicoGreen dsDNA quantification kit (Invitrogen). The amplicon pool then underwent a second purification step with Agencourt AMPure-XP beads (Beckman Coulter, Brea, CA, USA) using an adapted heat-denaturation protocol (Roche). Emulsion PCR and emulsion breaking were performed following protocols of Roche and pyrosequencing was performed on a 454 GS FLX+ sequencer by IMGM Laboratories, Planegg, Germany.

### Analysis of sequencing data

Initial quality ﬁltering of the raw pyrosequencing reads was done by using the automated amplicon pipeline of the GS Run Processor with the LongAmplicon3 filter (Roche). Sequences were then de-multiplexed to separate MID barcodes (Pilloni *et al*. 2012), initial quality trimming was done in GREENGENES; using the TRIM function with the default settings (DeSantis *et al*. 2006). Trimmed sequences were uploaded and analyzed via the NGS analysis pipeline of the SILVA rRNA gene database project (SILVAngs 1.3) (Quast *et al*. 2013). Reads were aligned using the SILVA Incremental Aligner (SINA SINA v1.2.10 for ARB SVN (revision 21008)) (Pruesse, Peplies and Glöckner 2012) against the SILVA SSU rRNA SEED and quality controlled (Quast *et al*. 2013). Reads shorter than 50 aligned nucleotides or below 40 alignment score, reads with more than 2% of ambiguities or more than 2% of homopolymers were excluded from the downstream processing. Dereplication and clustering of the unique reads into operational taxonomic units (OTUs) was done by using cd-hit-est (version 3.1.2) (Li and Godzik 2006) running in accurate mode, ignoring overhangs and applying identity criteria of 1.00 and 0.98, respectively. The classification of the OTUs was performed by a local nucleotide BLAST search against the non-redundant version of the SILVA SSU Ref dataset (release 123; http://www.arb-silva.de) using blastn (version 2.2.30) with standard settings (Camacho *et al*. 2009). Weak BLAST hits (below 93%) or reads without any BLAST hits remained unclassified and were assigned to the metagroup “No Relative". For downstream data handling, relative abundances were selected from the SILVAngs pipeline output. OTUs with less than 1% relative abundance were summarized in a composite “<1%” group. Selected amplicon contigs have been deposited at GenBank under the accession numbers KY499472 to KY499476. All sequencing read raw data are deposited at the SRA under the project accession numbers SAMN07673532-SAMN07673540.

### Cloning, sequencing and phylogenetic analysis

C23O amplicons generated with the primer set XYLE3F/XYLE3R were cloned and sequenced (Táncsics *et al*. 2013) from the initial sediment sample, as well as from selected “heavy” and “light” DNA fractions of the day 3 ^13^C-toluene SIP gradient. Selected terminal restriction fragments (T-RFs) predicted *in silico* for representative clones were verified *in vitro*. Phylogenetic trees were reconstructed from sequence data using neighbor-joining as described (Táncsics *et al*. 2013). Sequences generated by cloning were deposited with GenBank and can be found under the accession numbers KY440386 – KY440395.

## RESULTS

### Exposure of sediments to ^13^C-toluene

Rapid depletion of toluene was observed in all enrichments (Fig. S1, Supporting Information) under simultaneous consumption of oxygen (data not shown). Roughly 70% of the toluene was depleted from the biotic enrichments after 3 d of incubation, while its concentration was under the detection limit by the seventh day of incubation (Fig. S1, Supporting Information). The abiotic loss from control incubations was marginal. Enrichments incubated for seven days received ∼7.8 mL of oxygen during the incubation. According to Wiedemeier et al. (1999) this amount of oxygen may be sufficient for the complete removal of 4.7 × 10^−5^ mol toluene (a concentration of ∼1 mM) present in the enrichments through biodegradation. Accordingly, consumption of oxygen considerably slowed by the end of the experiment, when toluene was depleted, as the oxygen injected on the 6th day of incubation was not completely consumed a day later (data not shown).

### Identification of labeled bacteria

**Figure 1.**
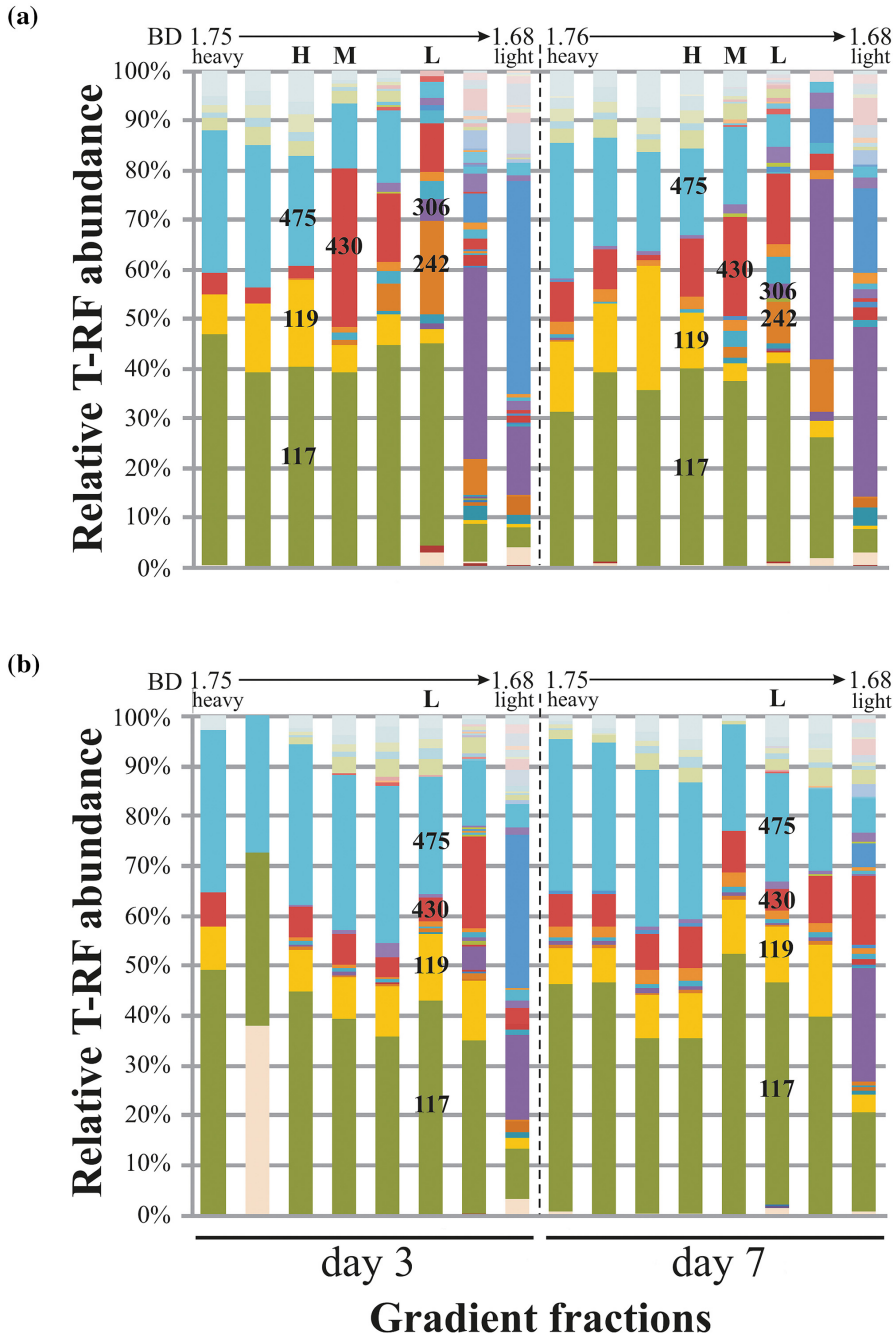
Abundance of bacterial 16S rRNA gene T-RFs across density-resolved gradient fractions of DNA from enrichments amended with (**A**) ^13^C or (**B**) ^12^C-toluene. Representative heavy (H), medium (M) and light (L) DNA fractions were selected and subjected to amplicon sequencing. Identified characteristic T-RFs (bp) mentioned in the text are indicated. BD, CsCl buoyant density (g mL^−1^).

Two time points were selected for the detection of labeled DNA by isopycnic centrifugation of extracts from single microcosms: day 3, where considerable degradation activity was suggested, and day 7, after toluene was depleted in the enrichments. At both time points, clear shifts in buoyant density (BD) compared to respective ^12^C-control DNA was observed in SIP gradients (Fig. S2, Supporting Information). Bacterial 16S rRNA gene-targeted T-RFLP fingerprinting of density resolved DNA detected clear distinctions between heavy and light DNA fractions of ^13^C-gradients (Fig. 1). Heavy fractions of DNA from ^13^C-toluene sediments showed a dominance of the 117-, 119- and 475-bp T-RFs. Light DNA fractions were enriched in the 242- and 306-bp T-RFs, while the 117 bp T-RF was also abundant here. In-between, medium BD fractions showed a selection of the 430-bp T-RF, giving a distinct community pattern between heavy and light fractions. DNA fractions from ^12^C-control gradients were more similar over the entire BD range and were dominated mainly by two T-RFs: the 117- and 475-bp fragments.

**Figure 2.**
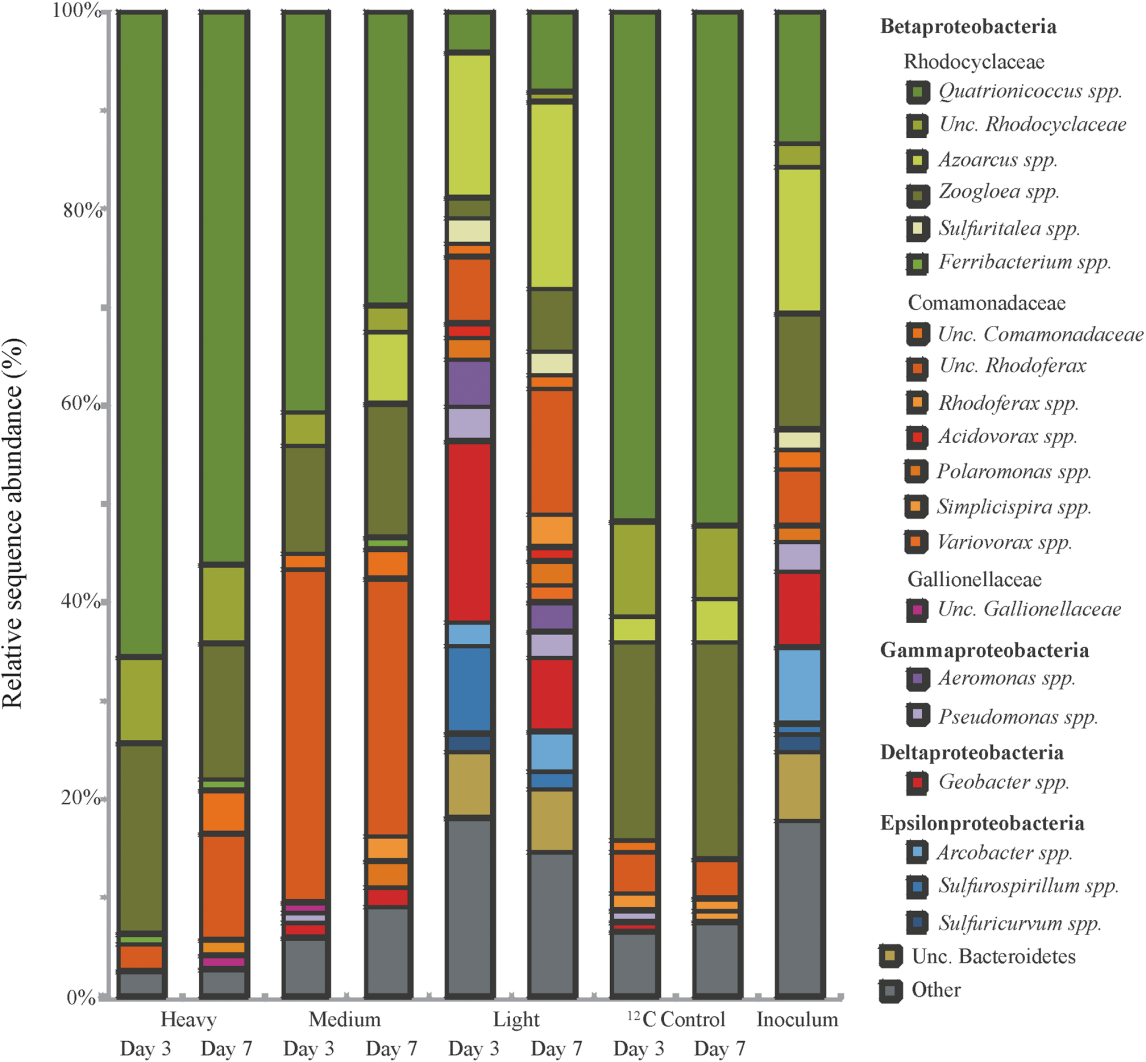
Relative read abundance of major taxa in bacterial pyrotag libraries of heavy, medium and light DNA fractions of ^13^C-toluene SIP gradients, representative light fractions of ^12^C-control gradients and the initial sediment inoculum. All genera contributing more than 1% abundance were depicted.

**Figure 3.**
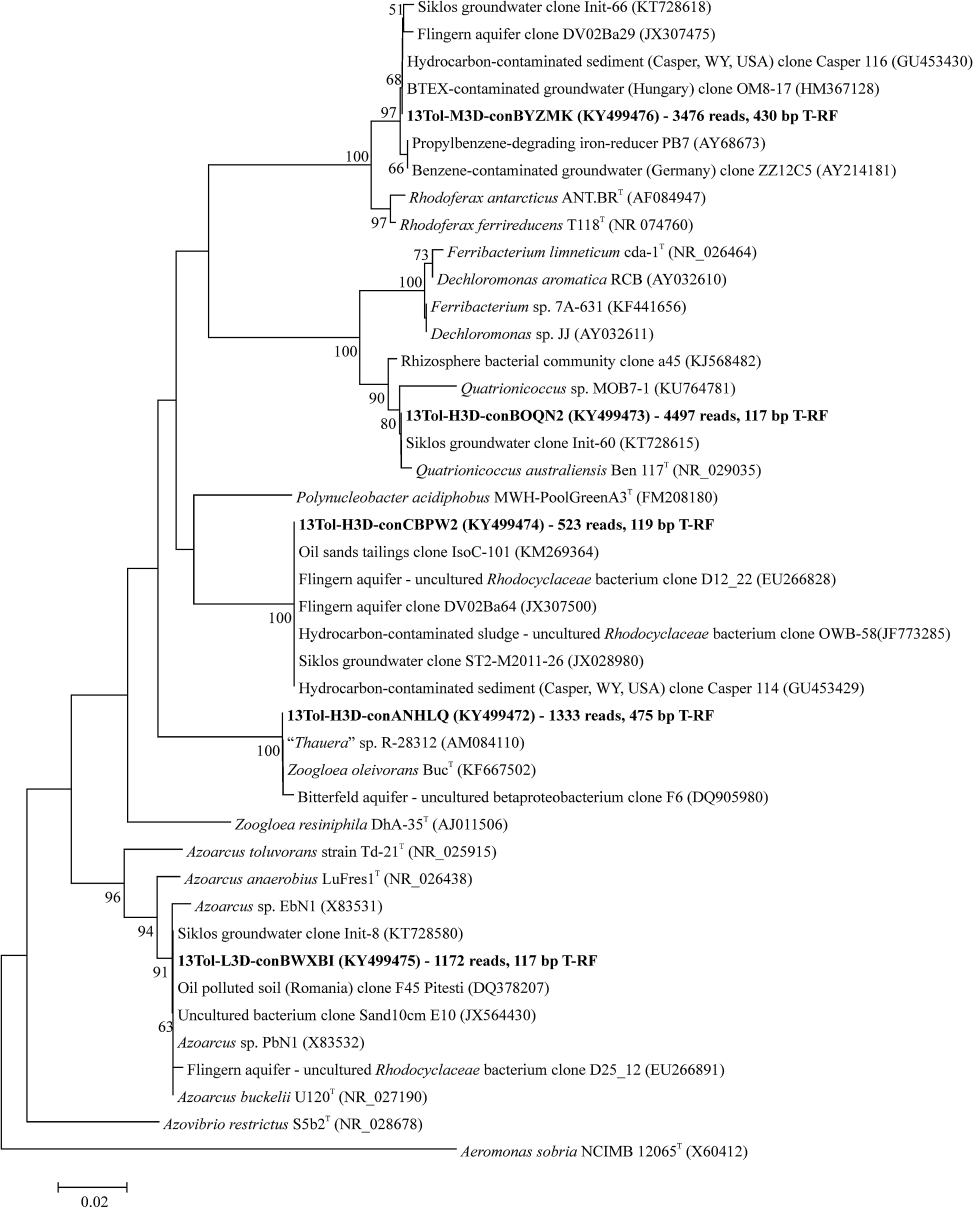
Phylogenetic placement of selected assembled OTU-level sequencing contigs (given in bold) of bacterial 16S rRNA gene amplicons from the SIP microcosms after 3 d. Contig naming indicates the DNA fraction (H - heavy, M - medium, L - light) as well as the incubation period (3D). Comprised total reads and predicted T-RFs (bp) are also indicated. T-RFs were predicted from sequence data, but are given as T-RFs actually measured in electropherograms, as first verified by Táncsics (2013). Bacterial lineages identified as key toluene degraders in SIP are highlighted in grey. GenBank accession numbers are also indicated. The tree was constructed using the neighbor-joining methods with Kimura’s two-parameter calculation model. Bootstrap values are shown as percentages of 1000 replicates; only values over 50% are shown. The 16S rDNA sequence of the gammaprotebacterium *Aeromonas sobria* (detected in the “light” DNA fractions) was used as outgroup. Scale bar, 0.02 substitutions per nucleotide position.

Bacterial 16S rRNA gene amplicons were sequenced from heavy, medium and light gradient fractions of ^13^C-gradients at both time points, as well as for ^12^C-control gradients and non-density resolved DNA from the initial sediment (Fig. 2, Table S1, Supporting Information). Libraries from heavy DNA after 3 and 7 d appeared especially enriched in reads affiliated to *Quatrionicoccus* spp., *Zoogloea* spp., as well as other uncultured *Rhodocyclaceae*. In combination with T-RFs previously reported for bacterial rRNA genes from the same site (Táncsics *et al*. 2013) the affiliation of T-RFs detected across gradients was thus possible (Fig. 3). The 117-bp T-RF in the “heavy” fractions represented amplicons affiliated to *Quatrionicoccus* spp (up to ∼60% read abundance in heavy DNA). The 119-bp and 475-bp T-RFs were linked to *Zoogloea* spp. and a yet uncultured member of the *Rhodocyclaceae*, respectively. In contrast, sequences represented by the 117-bp T-RF in the light fractions appeared mainly affiliated to *Azoarcus* spp. Thus, 16S rRNA gene sequencing resolved labeled and unlabeled bacterial populations apparently represented by the same (117-bp) T-RF. Besides, the 242- and 306-bp T-RFs detected in light fractions represented amplicons affiliated to *Geobacter* spp. and the *Bacteroidetes*. The 430-bp T-RF enriched in intermediate fractions represented reads related to *Rhodoferax* spp.

Furthermore, amplicon sequencing revealed that intermediate DNA fractions were still highly dominated by *Rhodocyclaceae*, but reads within the *Comamonadaceae* were also observed, mostly affiliated to the yet uncultured lineage of genus *Rhodoferax*. In the light DNA fractions the abundance of reads within the *Betaproteobacteria* decreased while sequences affiliated to the *Gammaproteobacteria* (*Aeromonas* and *Pseudomonas* spp.), *Deltaproteobacteria* (*Geobacter* spp.), *Epsilonproteobacteria* (*Arcobacter* and *Sulfurospirillum* spp.) as well as *Bacteroidetes* consistently became more abundant in both ^13^C-gradients (Fig. 2, Table S1, Supporting Information).

### Subfamily I.2.C-type C23O genes detected in SIP gradient fractions

**Figure 4.**
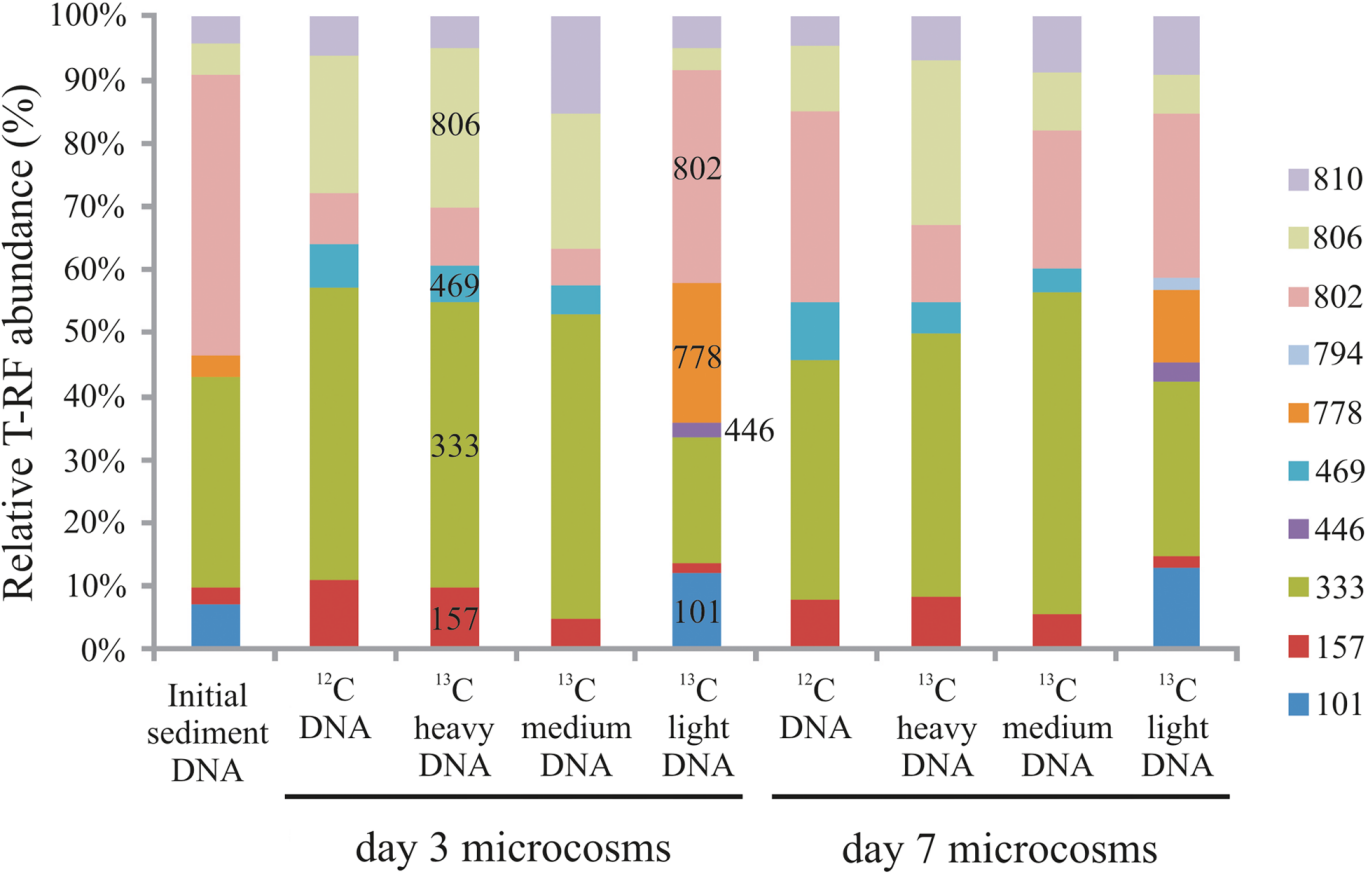
T-RFLP fingerprinting of subfamily I.2.C-type C23O genotypes from heavy, medium and light DNA fractions of ^13^C-toluene SIP gradients, representative light fractions of ^12^C-control gradients and the initial sediment sample. Characteristic T-RFs (bp) mentioned in the text are indicated.

The diversity of I.2.C-type C23O genes at the Siklós site has been investigated previously (Táncsics *et al*. 2012, 2013). However, the majority of the genotypes detected could not be affiliated to known bacterial degraders of BTEX compounds at that time. To address this, a T-RFLP fingerprinting assay targeting I.2.C-type C23O genes was applied to screen density resolved C23O genotypes. In contrast to 16S rRNA gene targeted T-RFLP fingerprinting, heavy and intermediate DNA fractions showed similar C23O fingerprints (Fig. 4). Dominant C23O T-RFs in heavy fractions were the 333- and 806-bp T-RFs at both time points, of which the first was also highly abundant in the inoculum. Further minor T-RFs (157- and 469-bp) were also enriched in heavy fractions. The dominant T-RF in the light fractions was at 802-bp, highly abundant also in the initial inoculum. Furthermore, C23O T-RFs at 778-, 101- and 446-bp were exclusively detectable in light DNA.

**Figure 5.**
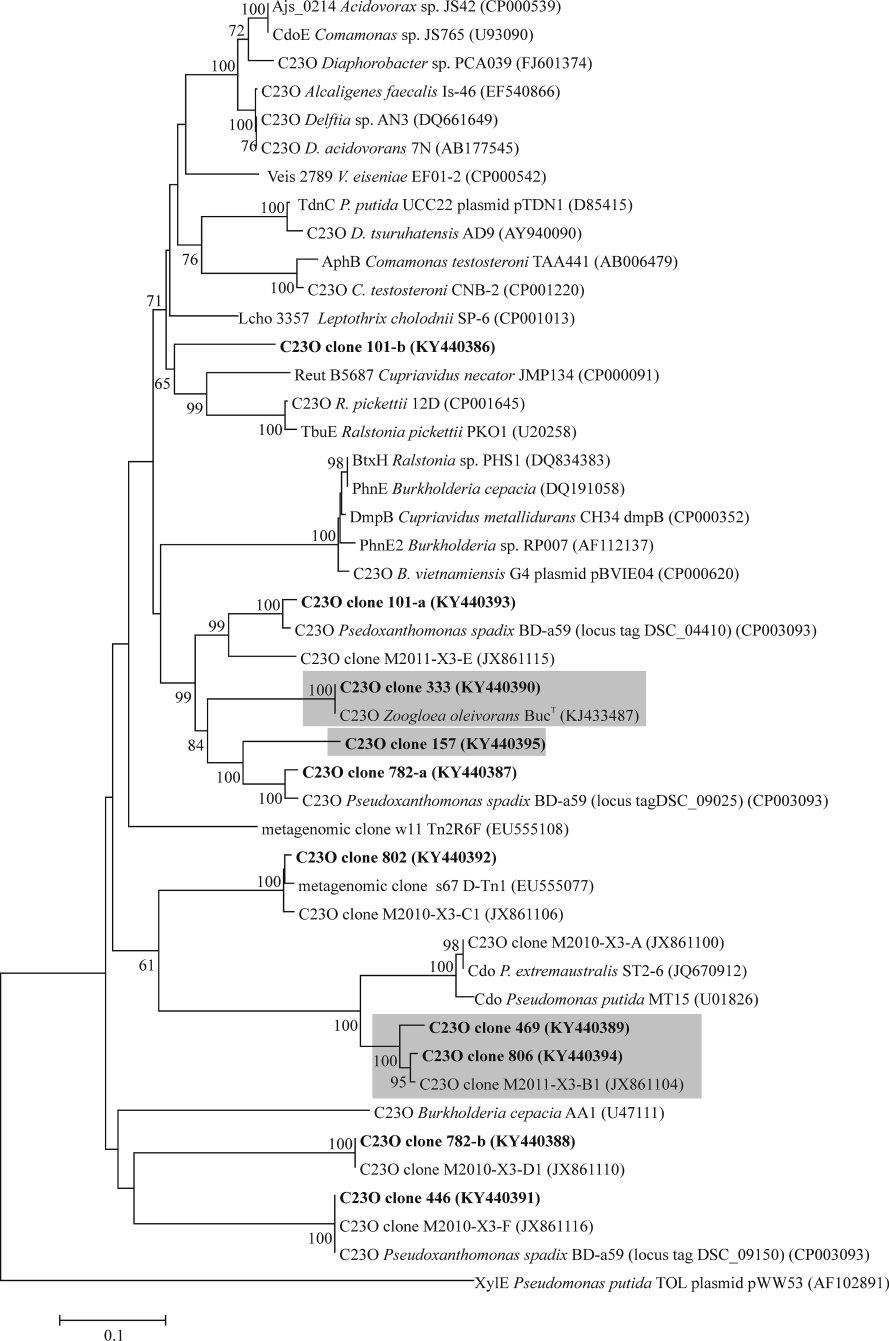
Neighbor-joining tree showing the phylogenetic placement of subfamily I.2.C-type C23O gene clones retrieved from the initial sediment DNA, heavy and light DNA fractions of day 3 ^13^C-toluene SIP gradient. Clones from this study are in bold, GenBank accession numbers are indicated. Clone naming includes measured T-RF lengths (*Alu*I digestion). Clones dominantly or exclusively found in the heavy DNA fractions are highlighted in grey. Bootstrap values are shown as percentages of 1000 replicates; only values over 50% are shown. The subfamily I.2.A-type C23O gene of TOL plasmid pWW53 was used as outgroup. Scale bar, 0.1 substitutions per nucleotide position.

To identify these T-RFs, clone libraries of C23O amplicons were generated and sequenced from the inoculum, as well as heavy and light DNA fractions of the day 3 microcosm. Thus, the 333-bp T-RF represented known C23O genes of *Zoogloea oleivorans* (Fig. 5), while the 806-bp T-RF represented a yet unaffiliated C23O genotype with low similarity (86% at the nucleotide level) to the *cdo* gene of *P. putida* MT15. Other minor T-RFs in heavy fractions represented unaffiliated C23O genes as well. In contrast, the 802-bp T-RF dominating the C23O gene pool in light DNA fractions, represented sequence types with high similarity (99%) to a yet unaffiliated, but heterologously characterized C23O gene (Brennerova *et al*. 2009). Furthermore, the 101-, 446- and 778-bp T-RFs represented three I.2.C-type C23O genes of *Pseudoxanthomonas spadix*. However, other amplicons related to yet unaffiliated C23O genes were also comprised in these fingerprinting peaks.

## DISCUSSION

Although the diversity of bacterial communities and subfamily I.2.C-type C23O gene pools at the Siklós site has been previously investigated (Táncsics *et al*. 2012, 2013), the affiliation of detected C23O genotypes and their possible role in oxic or hypoxic degradation processes remained unclear. The aim of this study was to address this by means of ^13^C-labelling in combination with fingerprinting and sequencing of 16S rRNA and I.2.C-type C23O gene amplicons from SIP gradients.

Toluene-degrading communities in site sediments were investigated at two time points of ^13^C labelling. A dominance of *Rhodocyclaceae*-related sequences was found in heavy DNA fractions. Especially, a *Quatrionicoccus*-related bacterium was thus identified as important hypoxic toluene degrader. The genus *Quatrionicoccus* contains only the type species *Q. australiensis*, which was isolated from activated sludge and is described as a strictly aerobic, Gram-negative coccus (Maszenan *et al*. 2002). The high abundance of this bacterium in the groundwater of the Siklós site has been noted earlier (Farkas *et al*. 2017). However, aromatic hydrocarbon degrading capability of the type strain has not been tested, and it is currently not available in culture collections. The two most closely related genera of *Quatrionicoccus* are *Ferribacterium* and *Dechloromonas* spp., the latter including *Dechloromonas aromatica*, a well-investigated aromatic hydrocarbon degrader (Coates *et al*. 2001). This is reported to degrade aromatic hydrocarbons using a dioxygenase-based pathway (not subfamily I.2.C C23O-based) under respiration of oxygen, chlorate or nitrate, giving rise to speculations about cryptic catabolic pathways at the interphase of aerobic and anaerobic metabolism (Salinero *et al*. 2009; Weelink, van Eekert and Stams 2010; Lueders 2017).

The second most abundant labeled degrader lineage detected in heavy DNA was *Zoogloea* spp. Screening of subfamily I.2.C C23O genes across gradient fractions indicated consistent labelling of *meta*-cleavage pathway encoding genes affiliated to *Zoogloea*. Members of this genus are primarily known for their floc-forming ability in sewage treatment plants, making them critical components of activated sludge processes (Shao *et al*. 2009). Within the genus, *Z. resiniphila* and *Z. oleivorans* have been described as degraders of petroleum hydrocarbons (Farkas *et al*. 2015; Monh *et al*. 1999). Jechalke *et al*. (2013) has investigated benzene degradation by a biofilm community in an aerated groundwater treatment pond. rRNA-SIP revealed a prominent role of *Zoogloea*-related degraders in the system. The present study substantiates an important role of these aromatic hydrocarbon degraders in oxic or micro-oxic groundwater environments.

The second most abundant C23O genotype detected in heavy DNA was the as-yet unidentified catabolic gene lineage represented by the 806 bp T-RF. The high abundance and marked enrichment of this gene in ^13^C-labelled DNA suggests that it could be affiliated to one of the dominating degraders identified in labelled 16S rRNA genes. It is tempting to speculate that this C23O genotype could actually be hosted by the *Quatrionicoccus*-relatives, however also other scenarios cannot be excluded, since degradation of toluene by these bacteria must not essentially involve catabolic pathways via C23O. Also, we have previously tentatively affiliated (Táncsics *et al*. 2013) the 806-bp T-RF C23O phylotype to the yet unidentified *Rhodocyclaceae*-related 16S sequences which were also found, albeit at much lower abundance, in heavy DNA fractions. The closest relative of these bacteria is *Polynucleobacter acidiphobus* (∼95.5% 16S rDNA similarity). However, as long as isolates of either of these *Rhodocyclaceae*-related degraders or of *Quatrionicoccus* spp. are not available, these interpretations must clearly be cautioned. Alternatively, metagenomics of single-cell approaches (Blainey 2013; Rinke *et al*. 2014) may also help to resolve this dilemma.

Besides the abundant peaks of fully ^13^C-labelled DNA detected in heavy gradient fractions, a distinct community was also observed in intermediate gradient fractions. Here, 16S rRNA reads of the genus *Rhodoferax* were consistently enriched, *Rhodoferax ferrireducens* being their closest relative (∼96% 16S rDNA similarity). These bacteria have been frequently reported from oxygen-limited or anaerobic subsurface environments contaminated with petroleum hydrocarbons (Callaghan *et al*. 2010; Aburto and Peimbert 2011; Táncsics *et al*. 2010, 2013; Larentis, Hoermann and Lueders 2013; Tischer *et al*. 2013). Moreover, previous SIP studies have indicated a role of this lineage in the aerobic degradation of phenantrene and naphthalene (Jeon *et al*. 2003; Martin *et al*. 2012). Results of our present study suggest that these bacteria may also have a role in the degradation of toluene, although labeling was not as apparent as for other dominating degraders. This could potentially be explained by the fact that certain *Rhodoferax* species grow very slowly (Kaden *et al*. 2014), and it was shown that *R. ferrireducens* is more adapted for high growth yields than rapid growth (Zhuang *et al*. 2011).

The main unlabeled lineages detected in the microcosms were affiliated to *Geobacter* and *Azoarcus* spp. Members of both genera are well known as anaerobic toluene degraders (Lueders 2017). While both may have originally been active in deeper oxygen-limited sediments at the site, they were clearly not active in our hypoxic microcosms. More surprisingly, reads affiliated to *Pseudomonas* spp. also remained unlabeled during SIP incubation. *P. putida* is one of the most widely utilized model organisms for the study of aerobic toluene degradation (Martínez-Lavanchy *et al*. 2010). On the other hand, *Pseudomonas*-affiliated subfamily I.2.C-type C23O genes, which could have enabled these bacteria to take part in the degradation of toluene under hypoxic conditions (Kukor and Olsen 1996), were not detected in the Siklós samples. It also has to be noted that *Pseudoxanthomonas spadix* (capable of degrading all BTEX-compounds) usually harbors three subfamily I.2.C-type C23O genes in its genome (Kim *et al*. 2008; Lee *et al*. 2012). All of them were detectable, but remained unlabeled in our study, just like the 16S rRNA genes of *Pseudoxanthomonas* spp. Nevertheless, toluene concentration in the microcosms was ∼1 mM, which can be toxic even for some toluene-degrading bacteria (Rabus *et al*. 1993) and could cause their inactivity as well.

The most dominant C23O genotype in the light fractions (802-bp T-RF) showed high similarity with metagenomic C23O clones retrieved by Brennerova *et al*. (2009) from jet-fuel contaminated soil. Functional genomics showed that the enzyme coded by this C23O genotype preferred 3-methylcatechol as substrate, an intermediate of aerobic toluene degradation. Nevertheless, bacteria harboring this C23O genotype were not labeled in our SIP microcosms. It is possible to speculate that these degraders could actually prefer nitrate as electron-acceptor under hypoxic conditions, while utilizing available oxygen for catabolic oxygenases (Wilson and Bouwer 1997). Since we did not add nitrate to the microcosms, and the fact the Siklós site is depleted in nitrate (Táncsics *et al*. 2013), such degraders may have remained inactive during our experiment.

In summary, this study shows that a notable diversity of degraders within the *Rhodocyclaceae* is active in hypoxic toluene degradation in sediments from the Siklós site. This includes previously unidentified degraders related to *Quatrionicoccus* spp., as well as their tentatively affiliated catabolic gene lineages. We also show that identified microaerobic toluene degraders mostly harbored subfamily I.2.C-type C23O genes, which may be of crucial importance for the degradation of aromatic hydrocarbons under oxygen-limited conditions. However, not all C23O genotypes were actually ^13^C-labelled, suggesting that ecophysiological fine-tuning, rather than catabolic repertoire contributes to niche definition between aerobic and hypoxic degraders of BTEX compounds in groundwater systems.

## Supplementary Material

Supplementary Data

## References

[bib1] Aburto A, Peimbert M Degradation of a benzene-toluene mixture by hydrocarbon-adapted bacterial communities. Ann Microbiol. 2011;61:553–62. 21949494 10.1007/s13213-010-0173-6 PMC3156334

[bib2] Balcke GU, Wegener S, Kiesel B Kinetics of chlorobenzene biodegradation under reduced oxygen levels. Biodegradation. 2008;19:507–18. 17934786 10.1007/s10532-007-9156-0

[bib2_364_1527066896466] Benedek T, Táncsics A, Szabó I Polyphasic analysis of an Azoarcus-Leptothrix-dominated bacterial biofilm developed on stainless steel surface in a gasoline-contaminated hypoxic groundwater. Environ Sci Pollut Res Int. 2016;23:9019–35. 26825521 10.1007/s11356-016-6128-0

[bib3] Blainey PC The future is now: single-cell genomics of bacteria and archaea. FEMS Microbiol Rev. 2013;37:407–27. 23298390 10.1111/1574-6976.12015 PMC3878092

[bib5] Brennerova MV, Josefiova J, Brenner V Metagenomics reveals diversity and abundance of meta-cleavage pathways in microbial communities from soil highly contaminated with jet fuel under air-sparging bioremediation. Environ Microbiol. 2009;11:2216–27. 19575758 10.1111/j.1462-2920.2009.01943.x PMC2784041

[bib4] Bruns A, Cypionka H, Overmann J Cyclic AMP and acyl homoserine lactones increase the cultivation efficiency of heterotrophic bacteria from the central Baltic Sea. Appl Environ Microbiol. 2002;68:3978–87. 12147499 10.1128/AEM.68.8.3978-3987.2002 PMC124024

[bib6] Callaghan AV, Davidova IA, Savage-Ashlock K Diversity of benzyl- and alkylsuccinate synthase genes in hydrocarbon-impacted environments and enrichment cultures. Environ Sci Technol. 2010;44:7287–94. 20504044 10.1021/es1002023

[bib7] Camacho C, Coulouris G, Avagyan V BLAST+: architecture and applications. BMC Bioinformatics. 2009;10:1–9. 20003500 10.1186/1471-2105-10-421 PMC2803857

[bib8] Coates JD, Chakraborty R, Lack JG Anaerobic benzene oxidation coupled to nitrate reduction in pure culture by two strains of *Dechloromonas*. Nature. 2001;411:1039–43. 11429602 10.1038/35082545

[bib9] Corteselli EM, Aitken MD, Singleton DR Rugosibacter aromaticivorans gen. nov., sp. nov., a novel bacterium within the family Rhodocyclaceae isolated from contaminated soil, capable of degrading aromatic compounds. Int J Syst Evol Microbiol. 2017;67:311–8. 27902243 10.1099/ijsem.0.001622 PMC5797942

[bib10] DeSantis TZ, Hugenholtz P, Larsen N Greengenes, a chimera-checked 16S rRNA gene database and workbench compatible with ARB. Appl Environ Microbiol. 2006;72:5069–72. 16820507 10.1128/AEM.03006-05 PMC1489311

[bib11] El-Naas MH, Acio JA, El Telib AE Aerobic biodegradation of BTEX: progresses and prospects. J Environ Chem Eng. 2014;2:1104–22.

[bib13] Farkas M, Szoboszlay S, Benedek T Enrichment of dissimilatory Fe(III)-reducing bacteria from groundwater of the Siklós BTEX-contaminated site (Hungary). Folia Microbiol. 2017;62:63–71. 27680983 10.1007/s12223-016-0473-8

[bib12] Farkas M, Táncsics A, Kriszt B *Zoogloea oleivorans* sp. nov., a floc-forming, petroleum hydrocarbon-degrading bacterium isolated from biofilm. Int J Syst Evol Microbiol. 2015;65:274–9. 25342113 10.1099/ijs.0.068486-0

[bib14] Jechalke S, Franchini AG, Bastida F Analysis of structure, function, and activity of a benzene-degrading microbial community. FEMS Microbiol Ecol. 2013;85:14–26. 23398624 10.1111/1574-6941.12090

[bib15] Jeon CO, Park W, Padmanabhan P Discovery of a bacterium, with distinctive dioxygenase, that is responsible for *in situ* biodegradation in contaminated sediment. Proc Natl Acad Sci USA. 2003;100:13591–6. 14597712 10.1073/pnas.1735529100 PMC263858

[bib16] Kaden R, Spröer C, Bexer D *Rhodoferax saidenbachensis* sp. nov., a psychrotolerant, very slowly growing bacterium within the family Comamonadaceae, proposal of appropriate taxonomic position of *Albidiferax ferrireducens* strain T118T in the genus *Rhodoferax* and emended description of the genus *Rhodoferax*. Int J Syst Evol Microbiol. 2014;64:1186–93. 24408525 10.1099/ijs.0.054031-0

[bib17] Karwautz C, Lueders T Impact of hydraulic well restoration on native bacterial communities in drinking water wells. Microbes Environ. 2014;29:363–9. 25273229 10.1264/jsme2.ME14035 PMC4262359

[bib18] Kim JM, Le NT, Chung BS Influence of soil components on the biodegradation of benzene, toluene, ethylbenzene, and o-, m-, and p-xylenes by the newly isolated bacterium *Pseudoxanthomonas spadix* BD-a59. Appl Environ Microbiol. 2008;74:7313–20. 18835999 10.1128/AEM.01695-08 PMC2592918

[bib19] Kukor JJ, Olsen RH Catechol 2,3-dioxygenases functional in oxygen-limited (hypoxic) environments. Appl Environ Microbiol. 1996;62:1728–40. 8633871 10.1128/aem.62.5.1728-1740.1996 PMC167947

[bib20] Kunapuli U, Lueders T, Meckenstock RU The use of stable isotope probing to identify key iron-reducing microorganisms involved in anaerobic benzene degradation. ISME J. 2007;1:643–53. 18043671 10.1038/ismej.2007.73

[bib21] Larentis M, Hoermann K, Lueders T Fine-scale degrader community profiling over an aerobic/anaerobic redox gradient in a toluene-contaminated aquifer. Environ Microbiol Rep. 2013;5:225–34. 23584966 10.1111/1758-2229.12004

[bib22] Lee SH, Jin HM, Lee HJ Complete genome sequence of the BTEX-degrading bacterium *Pseudoxanthomonas spadix* BD-a59. J Bacteriol. 2012;194:544. 22207748 10.1128/JB.06436-11 PMC3256636

[bib23] Li W, Godzik A Cd-hit: a fast program for clustering and comparing large sets of protein or nucleotide sequences. Bioinformatics. 2006;22:1658–9. 16731699 10.1093/bioinformatics/btl158

[bib26] Lueders T, Kindler R, Miltner A Identification of bacterial micropredators distinctively active in a soil microbial food web. Appl Environ Microbiol. 2006;72:5342–8. 16885285 10.1128/AEM.00400-06 PMC1538704

[bib24] Lueders T DNA- and RNA-based stable isotope probing of hydrocarbon degraders. In: McGenity TJ, Timmis K, Nogales B (eds). Hydrocarbon and Lipid Microbiology Protocols. Springer Protocols Handbooks. Berlin, Heidelberg: Springer, 2015, 181–97.

[bib25] Lueders T The ecology of anaerobic degraders of BTEX hydrocarbons in aquifers. FEMS Microbiol Ecol. 2017;93DOI: 10.1093/femsec/fiw220. PMC5400083 27810873

[bib27] Martin F, Torelli S, Le Paslier D Betaproteobacteria dominance and diversity shifts in the bacterial community of a PAH-contaminated soil exposed to phenantrene. Environ Pollut. 2012;162:345–53. 22243884 10.1016/j.envpol.2011.11.032

[bib28] Martínez-Lavanchy PM, Müller C, Nijenhuis I High stability and fast recovery of expression of the TOL plasmid-carried toluene catabolism genes of *Pseudomonas putida* mt-2 under conditions of oxygen limitation and oscillation. Appl Environ Microbiol. 2010;76:6715–23. 20709833 10.1128/AEM.01039-10 PMC2953008

[bib29] Maszenan AM, Seviour RJ, Patel BKC *Quadricoccus australiensis* gen. nov., sp. nov., a β-proteobacterium from activated sludge biomass. Int J Syst Evol Microbiol. 2002;52:223–8. 11858148 10.1099/00207713-52-1-223

[bib30] Monh WW, Wilson AE, Bicho P Physiological and phylogenetic diversity of bacteria growing on resin acids. Syst Appl Microbiol. 1999;22:68–78. 10188280 10.1016/S0723-2020(99)80029-0

[bib31] Parales RE Hydrocarbon degradation by betaproteobacteria. In: Timmis K (ed). Handbook of Hydrocarbon and Lipid Microbiology. Berlin, Heidelberg: Springer, 2010, 1715–24.

[bib32] Pilloni G, Granitsiotis MS, Engel M Testing the limits of 454 pyrotag sequencing: reproducibility, quantitative assessment and comparison to T-RFLP fingerprinting of aquifer microbes. PLoS One. 2012;7:e40467. 22808168 10.1371/journal.pone.0040467 PMC3395703

[bib33] Pilloni G, von Netzer F, Engel M Electron acceptor-dependent identification of key anaerobic toluene degraders at a tar-oil-contaminated aquifer by Pyro-SIP. FEMS Microbiol Ecol. 2011;78:165–75. 21385190 10.1111/j.1574-6941.2011.01083.x

[bib34] Pruesse E, Peplies J, Glöckner FO SINA: Accurate high-throughput multiple sequence alignment of ribosomal RNA genes. Bioinformatics. 2012;28:1823–9. 22556368 10.1093/bioinformatics/bts252 PMC3389763

[bib35] Quast C, Pruesse E, Yilmaz P The SILVA ribosomal RNA gene database project: improved data processing and web-based tools. Nucleic Acids Res. 2013;41:590–6. 10.1093/nar/gks1219 PMC3531112 23193283

[bib36] Rabus R, Nordhaus R, Ludwig W Complete oxifation of toluene under strictly anoxic conditions by a new sulfate-reducing bacterium. Appl Environ Microbiol. 1993;59:1444–51. 7686000 10.1128/aem.59.5.1444-1451.1993 PMC182102

[bib37] Rinke C, Lee J, Nath N Obtaining genomes from uncultivated microorganisms using FACS-based single-cell genomics. Nat Protoc. 2014;9:1038–48. 24722403 10.1038/nprot.2014.067

[bib38] Salinero KK, Keller K, Feil WS Metabolic analysis of the soil microbe *Dechloromonas aromatica* str. RCB: indications of a surprisingly complex life-style and cryptic anaerobic pathways for aromatic degradation. BMC Genomics. 2009;10:351. 19650930 10.1186/1471-2164-10-351 PMC2907700

[bib39] Shao Y, Chung BS, Lee SS *Zoogloea caeni* sp. nov., a floc-forming bacterium isolated from activated sludge. Int J Syst Evol Microbiol. 2009;59:526–30. 19244434 10.1099/ijs.0.65670-0

[bib43] Tischer K, Kleinsteuber S, Schleinitz KM Microbial communities along biochemical gradients in a hydrocarbon-contaminated aquifer. Environ Microbiol. 2013;15:2603–15. 23809669 10.1111/1462-2920.12168

[bib42] Táncsics A, ,Farkas M, Szoboszlay S One-year monitoring of meta-cleavage dioxygenase gene expression and microbial community dynamics reveals the relevance of subfamily I.2.C. extradiol dioxygenases in hypoxic, BTEX-contaminated groundwater. Syst Appl Microbiol. 2013;36:339–50. 23706914 10.1016/j.syapm.2013.03.008

[bib40] Táncsics A, Szabó I, Baka I Investigation of catechol 2,3-dioxagenase and 16S rRNA gene diversity in hypoxic, petroleum hydrocarbon contaminated groundwater. Syst Appl Microbiol. 2010;33:398–406. 20970942 10.1016/j.syapm.2010.08.005

[bib41] Táncsics A, Szoboszlay S, Szabó I Quantification of subfamily I.2.C catechol 2,3-dioxygenase mRNA transcripts in groundwater samples of an oxygen-limited BTEX-contaminated site. Environ Sci Technol. 2012;46:232–40. 22091737 10.1021/es201842h

[bib44] Weelink SAB, van Eekert MHA, Stams AJM Degradation of BTEX by anaerobic bacteria: physiology and application. Rev Environ Sci Biotechnol. 2010;9:359–85.

[bib45] Wiedemeier TH, Rifai HS, Newell CJ Natural Attenuation of Fuels and Chlorinated Solvents in the Subsurface. New York: Wiley, 1999.

[bib46] Wilson LP, Bouwer EJ Biodegradation of aromatic compounds under mixed oxygen/denitrifying conditions: a review. J Ind Microbiol Biotechnol. 1997;18:116–30. 9134760 10.1038/sj.jim.2900288

[bib1_991_1527066110806] Winderl C, Penning H, von Netzer F DNA-SIP identifies sulfate-reducing *Clostridia*as important toluene degraders in tar-oil-contaminated aquifer sediment. ISME J. 2010;4:1314–25. 20428224 10.1038/ismej.2010.54

[bib48] Zhang L, Lueders T Micropredator niche differentiation between bulk soil and rhizosphere of an agricultural soil depends on bacterial prey. FEMS Microbiol Ecol. 2017;93, DOI: 10.1093/femsec/fix103. 28922803

[bib49] Zhuang K, Izallalen M, Mouser P Genome-scale dynamic modeling of the competition between *Rhodoferax* and *Geobacter* in anoxic subsurface environments. ISME J. 2011;5:305–16. 20668487 10.1038/ismej.2010.117 PMC3105697

