# Supplementary material for: Stable isotope probing of hypoxic toluene degradation at the Siklós aquifer reveals prominent role of Rhodocyclaceae
Source: FEMS Microbiol Ecol. 2018 May 14;94(6):fiy088. doi: 10.1093/femsec/fiy088 (PMC5972620; doi:10.1093/femsec/fiy088)
Supplement: Supplementary Data [file fiy088_supp.docx]

**Supplementary Data**

**Fig. S1.** Time course of toluene concentrations in comparative ^12^C- and ^13^C-microcosms after 3 and 7 d of incubation. Means of triplicate microcosms are given (with standard error).

**
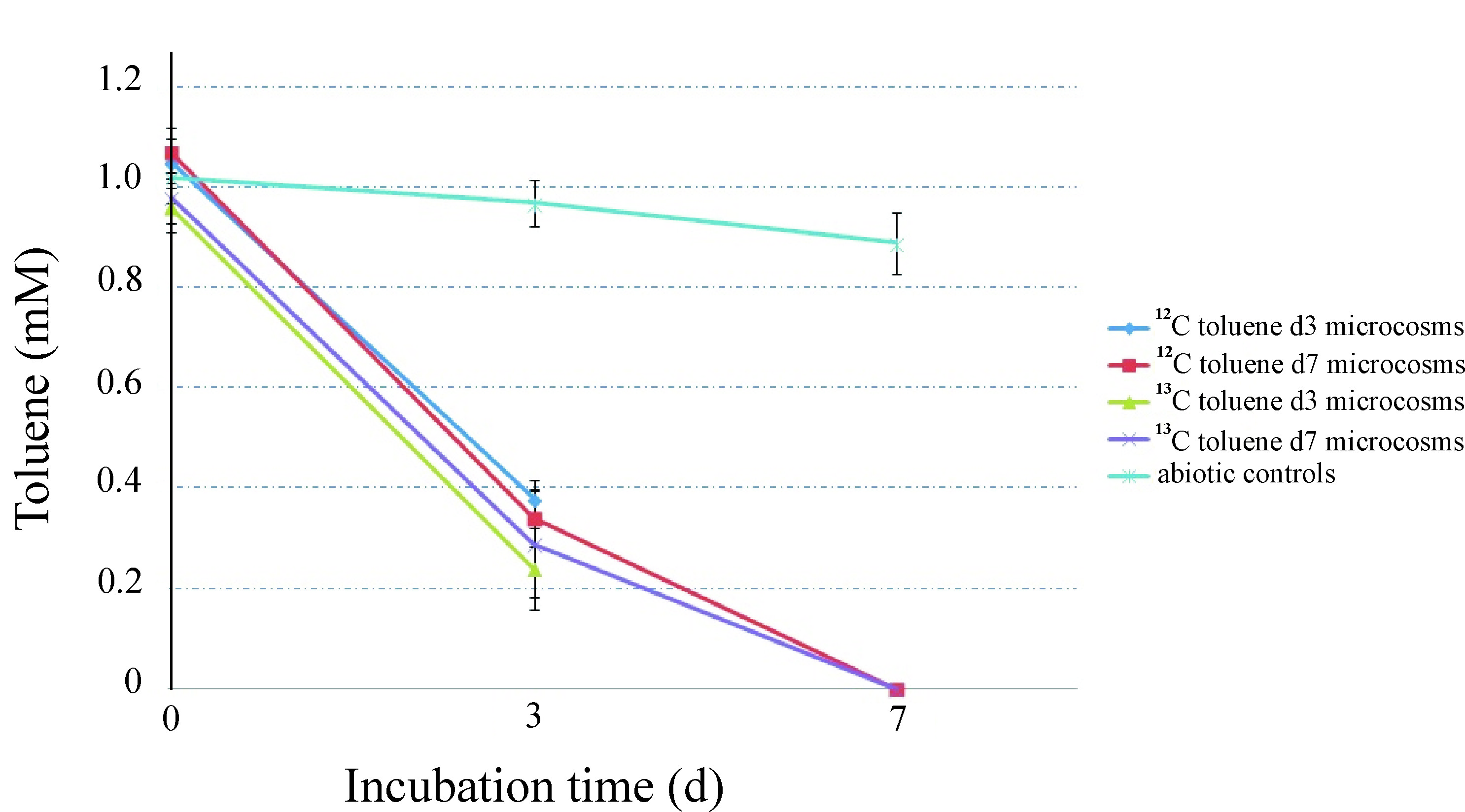
**

**Fig. S2.** Quantitative profiles of DNA distribution, measured via 16S rRNA gene qPCR, in comparative SIP gradients of DNA from microcosms amended with unlabelled (^12^C) toluene or ^13^C_7_-toluene.

**
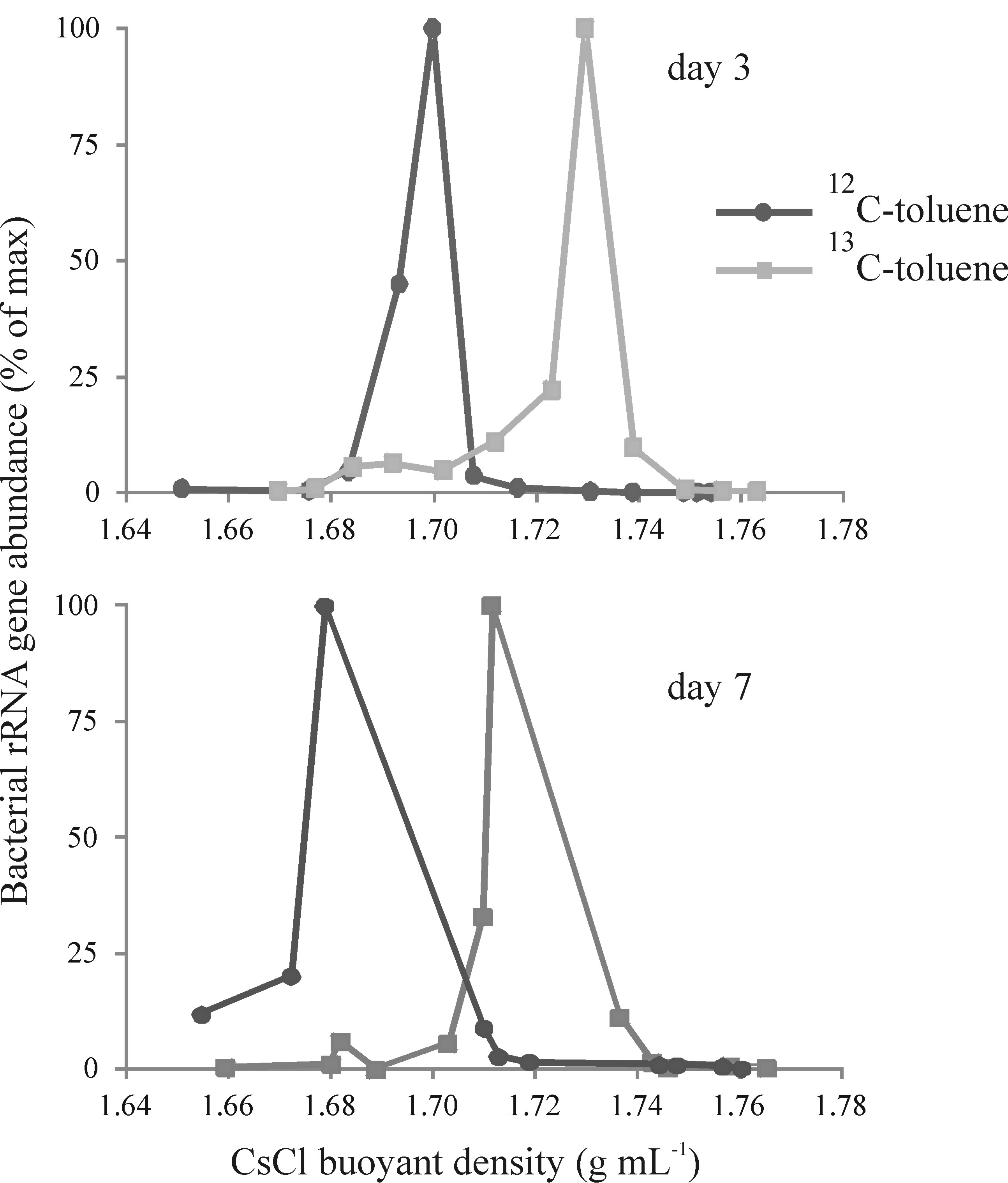
**

**Table S1. Phylogenetic affiliation and percentage abundance of trimmed reads produced in pyrosequencing of bacterial 16S rRNA gene fragments retrieved from the initial sediment DNA and from “heavy”, “medium” and “light” DNA fractions of ^13^C-toluene SIP gradients, representative light fractions of ^12^C-control gradients and the initial sediment sample.**

| **Phylogenetic affiliation** | Initial sediment (5118 reads) | **day 3 DNA fractions** | | | | **day 7 DNA fractions** | | | | T-RF*  [bp] |
| --- | --- | --- | --- | --- | --- | --- | --- | --- | --- | --- |
|  |  | SIP gradient fractions | | | ^12^C control (7145 reads) | SIP gradient fractions | | | ^12^C control (6454 reads) |  |
|  |  | heavy (6869 reads) | medium (10302 reads) | light (7927 reads) |  | heavy (8107 reads) | medium (9091 reads) | light (10247 reads) |  |  |
| ***Proteobacteria*** | **86.3** | **99.9** | **99.7** | **88.3** | **99.3** | **99.9** | **99.1** | **90.2** | **98.8** | NA |
| ***Betaproteobacteria*** | **58.4** | **99.4** | **96.1** | **41.7** | **95.4** | **99.2** | **93.6** | **65.7** | **95.6** | NA |
| ***Rhodocyclaceae*** | **45.9** | **95.1** | **56.1** | **26.6** | **86.0** | **79.6** | **55.8** | **39.8** | **76.6** | NA |
| Unc. *Rhodocyclaceae* | 2.4 | 8.8 | 3.4 | 0.5 | 9.7 | 8.0 | 2.7 | 1.0 | 7.5 | 119 |
| *Azoarcus* spp. | 14.8 | 0.1 | 0.8 | 14.8 | 2.6 | 0.4 | 7.3 | 19.0 | 4.4 | 117 |
| *Quatrionicoccus* spp. | 13.5 | 65.5 | 40.8 | 4.2 | 51.8 | 56.2 | 29.9 | 8.2 | 52.2 | 117 |
| *Zoogloea* spp. | 11.8 | 19.4 | 10.9 | 2.1 | 20.1 | 13.8 | 13.5 | 6.4 | 22.0 | 475 |
| *Dechloromonas* spp. | 0.2 | 0.3 | 0.2 | 0.8 | 0.3 | 0.1 | 0.3 | 0.8 | 0.1 | ND |
| *Sulfuritale*a spp. | 2.1 | 0.0 | 0.1 | 2.5 | 0.3 | 0.0 | 0.3 | 2.4 | 0.2 | ND |
| *Ferribacterium* spp. | 0.5 | 1.0 | 0.7 | 0.3 | 0.9 | 1.1 | 1.2 | 0.7 | 0.9 | ND |
| ***Comamonadaceae*** | **10.9** | **4.2** | **37.9** | **13.7** | **8.2** | **17.7** | **36.6** | **24.4** | **7.4** | NA |
| Unc. *Comamonad.* | 1.9 | 0.3 | 1.6 | 1.3 | 1.2 | 4.4 | 3.0 | 1.4 | 0.5 | ND |
| Unc. *Rhodoferax* | 5.7 | 2.8 | 33.7 | 6.8 | 4.2 | 10.7 | 26.2 | 12.7 | 4.0 | 430 |
| *Rhodoferax* spp. | 0.4 | 0.8 | 0.8 | 0.8 | 1.7 | 0.2 | 2.5 | 3.3 | 1.3 | ND |
| *Acidovorax* spp. | 0.5 | 0.2 | 0.4 | 1.5 | 0.3 | 0.5 | 0.8 | 1.4 | 0.2 | ND |
| *Polaromonas* spp. | 1.7 | 0.0 | 0.7 | 2.2 | 0.4 | 0.2 | 2.7 | 2.5 | 0.3 | ND |
| *Simplicispira* spp. | 0.2 | 0.0 | 0.5 | 0.4 | 0.3 | 1.6 | 0.6 | 0.8 | 1 | ND |
| *Variovorax* spp. | 0.2 | 0.0 | 0.0 | 0.2 | 0.0 | 0.0 | 0.2 | 1.7 | 0.0 | ND |
| ***Gallionellaceae*** | **0.0** | **0.0** | **1.2** | **0.0** | **0.0** | **1.5** | **0.0** | **0.0** | **0.0** | NA |
| Unc. *Gallionellaceae* | 0.0 | 0.0 | 1.2 | 0.0 | 0.0 | 1.5 | 0.0 | 0.0 | 0.0 | ND |
| ***Gammaproteobacteria*** | **4.6** | **0.3** | **1.0** | **9.5** | **1.7** | **0.2** | **1.0** | **6.7** | **0.6** | NA |
| *Aeromonas* spp. | 0.7 | 0.0 | 0.0 | 4.8 | 0.3 | 0.0 | 0.2 | 3.0 | 0.1 | ND |
| *Pseudomonas* spp. | 3.0 | 0.1 | 1.0 | 3.4 | 1.2 | 0.2 | 0.5 | 2.7 | 0.4 | ND |
| *Pseudoxanthomonas* spp. | 0.3 | 0.0 | 0.0 | 0.2 | 0.0 | 0.0 | 0.1 | 0.3 | 0.0 | ND |
| ***Deltaproteobacteria*** | **11.4** | **0.1** | **2.2** | **22.3** | **1.7** | **0.3** | **3.2** | **9.9** | **1.6** | NA |
| *Geobacter* spp. | 7.7 | 0.1 | 1.5 | 18.5 | 1.0 | 0.2 | 2.0 | 7.5 | 0.7 | 242 |
| *Desulfocapsa* spp. | 0.9 | 0.0 | 0.0 | 0.6 | 0.1 | 0.0 | 0.0 | 0.4 | 0.0 | ND |
| ***Epsilonproteobacteria*** | **10.9** | **0.1** | **0.3** | **13.2** | **0.0** | **0.1** | **0.7** | **6.6** | **0.0** | NA |
| *Arcobacter* spp. | 7.7 | 0.1 | 0.2 | 2.4 | 0.0 | 0.0 | 0.5 | 4.0 | 0.0 | ND |
| *Sulfurospirillum* spp. | 1.1 | 0.0 | 0.0 | 8.8 | 0.0 | 0.0 | 0.1 | 1.8 | 0.0 | ND |
| *Sulfuricurvum* spp. | 1.8 | 0.0 | 0.0 | 1.9 | 0.0 | 0.0 | 0.1 | 0.7 | 0.0 | ND |
| ***Firmicutes*** | **2.2** | **0.0** | **0.0** | **1.6** | **0.0** | **0.0** | **0.0** | **1.0** | **0.0** | NA |
| Unc. *Clostridiales* | 0.5 | 0.0 | 0.0 | 0.7 | 0.0 | 0.0 | 0.0 | 0.5 | 0.0 | ND |
| ***Bacteroidetes*** | **7.0** | **0.0** | **0.1** | **6.7** | **0.3** | **0.1** | **0.5** | **6.4** | **0.7** | 306 |
| **Other** | **4.5** | **0.1** | **0.3** | **3.4** | **0.7** | **0.1** | **0.9** | **2.4** | **1.2** | NA |

*Characteristic T-RFs were predicted for important lineages via assembled amplicon contigs, but are given as T-RFs actually observed in the electropherograms and verified in Táncsics et al., 2013. NA, not applicable for this level; ND, not detected for this lineage.
